# Recent Observations on Australian Bat Lyssavirus Tropism and Viral Entry

**DOI:** 10.3390/v6020909

**Published:** 2014-02-19

**Authors:** Dawn L. Weir, Edward J. Annand, Peter A. Reid, Christopher C. Broder

**Affiliations:** 1 Department of Microbiology, Uniformed Services University, Bethesda, MD 20814, USA; E-Mail: dawn.weir@usuhs.edu; 2 Equine Veterinary Surgeon, Randwick Equine Centre, Sydney 2031, Australia; E-Mail: edannand@gmail.com; 3 Equine Veterinary Surgeon, Brisbane, Queensland 4034, Australia; E-Mail: preidvet@bigpond.net.au

**Keywords:** rhabdovirus, Australian bat lyssavirus, rabies virus, viral entry, emerging, zoonosis, tropism, glycoprotein, endocytosis

## Abstract

Australian bat lyssavirus (ABLV) is a recently emerged rhabdovirus of the genus lyssavirus considered endemic in Australian bat populations that causes a neurological disease in people indistinguishable from clinical rabies. There are two distinct variants of ABLV, one that circulates in frugivorous bats (genus *Pteropus*) and the other in insectivorous microbats (genus *Saccolaimus*). Three fatal human cases of ABLV infection have been reported, the most recent in 2013, and each manifested as acute encephalitis but with variable incubation periods. Importantly, two equine cases also arose recently in 2013, the first occurrence of ABLV in a species other than bats or humans. Similar to other rhabdoviruses, ABLV infects host cells through receptor-mediated endocytosis and subsequent pH-dependent fusion facilitated by its single fusogenic envelope glycoprotein (G). Recent studies have revealed that proposed rabies virus (RABV) receptors are not sufficient to permit ABLV entry into host cells and that the unknown receptor is broadly conserved among mammalian species. However, despite clear tropism differences between ABLV and RABV, the two viruses appear to utilize similar endocytic entry pathways. The recent human and horse infections highlight the importance of continued Australian public health awareness of this emerging pathogen.

## 1. Discovery of Australian Bat Lyssavirus

The discovery of Hendra virus (HeV), a lethal paramyxovirus that emerged in Australia in 1994 [1], indirectly led to the discovery of Australian bat lyssavirus (ABLV), the first endemic lyssavirus isolated in Australia. In 1996, a retrospective study to identify the natural host of HeV was initiated. Serological evidence pointed to pteropid bats, also known as flying foxes, as the likely host reservoir of HeV [2]; indeed, HeV was subsequently isolated from these bats [3]. Brain tissue samples from two female black flying foxes collected from Ballina, Northern New South Wales (NSW) tested negative for HeV antibodies [4]. One sample, collected in 1996, was from a juvenile female black flying fox (*Pteropus alecto*) that was found under a tree and unable to fly; the other was a fixed paraffin-embedded tissue sample from a female black flying fox collected the previous year that had been euthanized and necropsied due to its unusually aggressive behavior [4]. Histological analyses of brain tissue from the 1996 bat showed severe nonsuppurative encephalitis. Encephalitis was mild in the 1995 bat brain sections but numerous cytoplasmic inclusion bodies, indicative of lyssavirus infection, were present and immunohistochemical analyses revealed the presence of lyssavirus nucleocapsid antigen throughout the brain of both bats [4]. Blood from the 1996 bat tested negative for anti-RABV neutralizing antibodies and virus could not be isolated by direct culture, but virus was subsequently isolated through intracerebral inoculation of kidney homogenate into weanling mice and further passaging in both mice and mouse neuroblastoma cells. The virus was shown to be neutralized by anti-RABV sera; however, the pattern of monoclonal antibody (mAb) binding of a panel of lyssavirus nucleoprotein (N) antibodies revealed that this lyssavirus was serologically distinct from RABV and other known lyssaviruses [4]. Sequence comparisons of the ABLV N protein and other lyssavirus N proteins showed that ABLV was most closely related to European bat lyssavirus-1 (EBLV1) and RABV with 93% and 92% amino acid identity, respectively [4,5]. Based on these results, as well as the anti-N mAb binding data, ABLV was designated as a new lyssavirus genotype.

A few months after the initial isolation of ABLV from the black flying fox, ABLV was isolated from an insectivorous microbat, the yellow-bellied sheathtail bat (*Saccolaimus flaviventris*) [6]. Nucleoprotein sequence comparisons revealed that the *Saccolaimus* N protein shared 96% amino acid homology with the *Pteropus* isolate and 90% amino acid homology with RABV. Phylogenetic analyses showed that *Saccolaimus* and *Pteropus* isolates separated into two clades and thus represented two distinct variants of ABLV: ABLVs and ABLVp for *Saccolaimus* and *Pteropus* variants, respectively. Additionally, mAb binding profiles were distinct for ABLVs and ABLVp and could be used to differentiate between the two ABLV variants [7]. 

## 2. Host Reservoirs and Geographic Distribution

**Figure 1:**
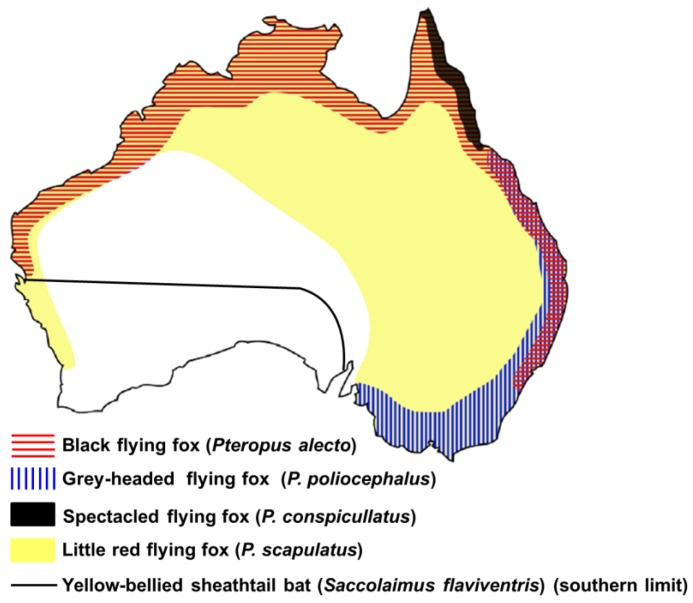
Distribution of ABLV host reservoir species. Adapted from [12,13,14].

Unlike RABV which has both terrestrial and bat reservoirs, only bats are known reservoirs of ABLV. Bats (order *Chiroptera*) are the second largest class of mammals with greater than 1,200 known species [8]. They are classified into two suborders, *Megachiroptera* (large Old World fruit bats; navigate by sight) and *Microchiroptera* (small bats, mostly insectivorous, found worldwide; navigate by echolocation). Australia has one family of Megachiropteran represented by five genera and 13 species [9]. ABLV has been isolated from all four species of flying foxes found on mainland Australia (suborder *Megachiroptera*, genus *Pteropus*): the black flying fox (*Pteropus alecto*), the grey-headed flying fox (*P. poliocephalus*), the little red flying fox (*P. scapulatus*), and the spectacled flying fox (*P. conspicillatus*) [6,10]. The combined host range of these four species extends from the west coast of Western Australia, through the Northern Territory, Queensland, New South Wales, and Victoria (Figure 1). The grey-headed flying fox is the only species restricted to Australia. The black flying fox, little red flying fox, and spectacled flying fox are also found in Papua New Guinea (PNG), and both the black flying fox and spectacled flying fox occur in parts of Indonesia [9]. Despite the extended geographic range of these three species outside of mainland Australia, ABLV has not been detected in PNG or Indonesian bat samples to date. However, the detection of neutralizing antibodies in six different bat species in the Philippines indicates the presence of ABLV or a closely related virus in Asia [11]. 

The Australian microchiroptera are much more diverse than the Australian megachiroptera, with six families containing 20 genera and 65 species identified on mainland Australia [9]. ABLV has been isolated from a single species of *Microchiroptera*, the yellow-bellied sheathtail bat (*Saccolaimus flaviventris*). The *Saccolaimus* strain is genetically distinct from the *Pteropus* strain [7]. The yellow-bellied sheathtail bat is widely distributed throughout mainland Australia (Figure 1) and is also native to PNG [9]. Despite the lack of additional virus isolations from other microbat species, serological evidence of ABLV infection has been reported in seven genera, representing five of the six families of microchiroptera found in Australia; all Australian bat species are considered as potential host reservoirs of ABLV [15].

### 2.1 Prevalence of ABLV

The prevalence of ABLV in healthy bats is estimated to be less than 1%. However, in sick, injured and/or orphaned flying foxes the prevalence of viral antigen as detected by the fluorescent antibody test (FAT) is typically 5%–10%, but may be as high as 17% or as low as 1%, depending on the species (16.9% in little red flying fox, 7.8% in black flying fox, 4.6% in grey-headed flying fox, and 1% in spectacled flying fox) [15]. The prevalence of ABLV is significantly higher in flying foxes displaying central nervous system (CNS) clinical signs. In one study, approximately 60% of sick or injured little red flying foxes with CNS symptoms tested positive for ABLV antigen [16]. ABLV seroprevalence in sick, injured and/or orphaned bats can be as high as 20% in flying foxes and 5% in microbats. However, injured yellow-bellied sheathtail microbats had an antibody prevalence as high as 62.5% [15].

## 3. Susceptible Species

### 3.1. Flying Foxes

There are numerous documented reports of observed clinical disease in flying foxes naturally infected with ABLV; two such incidents are described herein. In the first, a nine-day clinical disease course in an orphaned juvenile male black flying fox (*P. alecto*) approximately two to three weeks of age that was being raised by a volunteer wildlife animal caregiver was documented [17]. After six weeks of good health, the bat began to display clinical signs of neurological disease. On the first day of illness, the bat suddenly became aggressive towards its companion bat and began experiencing repeated muscle spasms during which it vocalized loudly. By the third day of illness, the bat was no longer aggressive and was only able to eat pulped food. Over the course of the next five days, the bat could no longer roost properly and was found lying down, face up. The bat developed worsening throat spasms, diarrhea, and began losing weight. On the ninth day of illness, its condition rapidly deteriorated and the bat died. Necropsy revealed nonsuppurative meningoencephalitis and numerous neurons contained eosinophilic inclusion bodies indicative of lyssavirus infection. FAT on fresh brain tissue confirmed ABLV infection; viral antigen was detected by immunoperoxidase staining in numerous sections of the brain, including the frontal cortex, hippocampus, brainstem, and cerebellum. Natural in utero infection with lyssaviruses is not known to occur, thus, the authors postulate that the bat was infected within the two to three weeks between its birth and when it was taken into care, indicating an incubation period of six to nine weeks [17]. In the second incident described by Warrilow *et al*. [18], a black flying fox with aggressive behavior was removed from the outside of a wire-mesh enclosure housing a colony of grey-headed flying foxes (*P. poliocephalus*) and euthanized; the bat tested positive for ABLV infection. One month later, a grey-headed flying fox from the colony began displaying signs of neurological disease; it was euthanized and was positive for ABLV infection. A common sequence from the variable noncoding intergenic region between the ABLV glycoprotein and polymerase was obtained for both bats, providing circumstantial evidence for natural cross-species bat-to-bat transmission [18]. 

### 3.2. Humans

ABLV has caused three fatal human infections; all occurred in Queensland. The first case occurred in late October of 1996. A 39-year-old female from Rockhampton developed pain and numbness in her left arm. She reported that she had received several scratches from flying foxes in her care in the preceding two to four week period, but had not been bitten [19,20]. She had also been caring for numerous other animals including dogs, cats, cockatoos, marsupials, and insectivorous bats. Over the next two to three days, she developed dizziness and vomiting followed by headache and fever and was admitted to the hospital. Intravenous treatment with broad spectrum antibiotics was started but her condition deteriorated and she developed diplopia, cerebellar signs, slurred speech, and had difficulty swallowing. By the 8th–10th days of illness, she had developed progressive weakness in all limbs and bilateral facial palsy; additionally, her level of consciousness began to fluctuate. By day eleven she was unresponsive and ventilator dependent; an electroencephalogram indicated diffuse encephalitis. Serum and cerebrospinal fluid (CSF) were analyzed by the Commonwealth Scientific and Industrial Research Organization (CSIRO) Australian Animal Health Laboratory (AAHL) in Geelong. The serum was positive for anti-lyssavirus antibodies and PCR to amplify part of the N gene of ABLV from the CSF resulted in a 250 base pair product, confirming that the patient was infected with ABLV. She was administered rabies immunoglobulin, but her condition continued to deteriorate and she died approximately 20 days after symptom onset [19,20]. It was later determined that the patient was likely bitten or scratched by a yellow-bellied sheathtail bat (*Saccolaimus flaviventris*) approximately 4.5 weeks prior to symptom onset [21]. Subsequent mAb binding profiles and genetic sequencing of virus isolated from fresh brain tissues of the patient confirmed that the *Saccolaimus* variant of ABLV was the likely source of infection [7].

The second fatal human ABLV infection occurred in late November 1998. A 37-year-old female was admitted to Mackay Base Hospital following a five-day history of fever, vomiting, pain in her left shoulder and left hand, and difficulty swallowing. She was well oriented, but febrile and was unable to fully open her mouth, had difficulty speaking and was drooling. When her throat was examined, spasmodic attempts to swallow ensued. Her condition rapidly deteriorated within the next 12 h, with increased agitation, and more frequent and severe muscle spasms; she was sedated and ventilated. It was soon discovered that she had a history of a bat bite and CSF, serum, and saliva were submitted for ABLV testing. By day two of hospitalization, she was no longer able to communicate or understand verbal commands and thereafter was ventilator-dependent. Two days later, a PCR product specific for ABLV was detected in the saliva and ABLV infection was confirmed four days later. On day 14 of hospitalization, artificial ventilation was ceased; she died 19 days after symptom onset [21]. Virus was isolated post-mortem from brain and spinal cord tissues. Sequencing of ABLV specific PCR product from infected cell cultures confirmed that the isolate was the *Pteropus* variant of ABLV. 

A detailed medical history revealed that the patient had attended a barbeque in late August of 1996 where a flying fox landed on the back of a small child. While removing the bat, the patient had been bitten on the base of her fifth left finger. She sought medical treatment two days after the bite and was administered antibiotics and tetanus toxoid. She returned to her doctor six months later, in early March, and inquired about a blood test for the “bat virus”. It was advised that she receive post-exposure prophylaxis (PEP) for RABV because of potential exposure to ABLV, but she declined treatment and succumbed to ABLV infection 27 months after initial exposure. The child and four other people exposed to the flying fox at the barbeque were administered PEP as soon as ABLVp infection was confirmed in the patient [21].

The third fatal human ABLV infection occurred in February, 2013. An eight-year-old boy from Long Island, Queensland was scratched by a flying fox (species unknown), but did not tell his parents about the bat encounter. He was admitted to Brisbane’s Mater Children’s Hospital approximately two months later, suffering with convulsions and abdominal pain and fever, followed by progressive brain problems and coma. On day 10 of hospitalization, ABLV infection was confirmed; the patient died 28 days after symptom onset [22,23].

### 3.3. Horses

The first confirmed cases of ABLV infection in horses occurred in May, 2013, the first spillover of ABLV into a species other than bats and humans [24,25]. An 18-month-old horse was observed to be off-color and displaying mild hind limb ataxia and mild pyrexia. The horse’s condition deteriorated over the next few days with increased ataxia, depression, hyperesthesia and trouble swallowing. Thirty six hours after symptom onset, the horse had difficulty standing and would not eat. Blood and nasal, oral and rectal swabs were collected and tested for HeV; PCR results were negative. The following day the horse began convulsing and was euthanized, 54 hours following clinical onset [26]. 

A second horse of the same age and having been kept in the same paddock demonstrated transient subtle symptoms of hind limb ataxia and demeanor alteration the day following the initial symptoms demonstrated by the first horse [26]. At this time the horse was normal on clinical examination and remained clinically normal for the following two days before again presenting similarly to his paddock mate with subtle hind limb ataxia. Twelve hours later the horse was severely depressed and demonstrated mydriasis, mild tachycardia, reduced gastrointestinal motility, pyrexia, and central nervous system symptoms, including head pressing, cervical ventroflexion, and severe ataxia. The horse was recumbent for part of the following day. HeV exclusion testing was again negative and the horse was euthanized following progressive deterioration as seen in the first case to lateral recumbancy and seizure 54 hours following the onset of clinical symptoms (Supplementary Video). Necropsy was only performed on the second horse and revealed severe sub-acute diffuse non-purulent encephalitis. The brain and spinal cord tissue was tested for ABLV and both PCR and FAT tests were positive. The PCR results indicated that the horse had been infected by the *Saccolaimus* strain of ABLV, suggesting that the horse had been exposed to ABLV through an infected microbat [26]. Remaining oral swab samples from the first horse were tested for ABLV and PCR confirmed that the horse had been infected by the *Saccolaimus* variant of ABLV [25].

### 3.4. Could Other Terrestrial Mammals be at Risk for ABLV Infection?

There have been no confirmed reports of ABLV infection in a species other than bats, humans, and horses despite known contacts of domestic dogs with infected flying foxes [27]. RABV and other bat lyssaviruses have caused disease in numerous mammalian species (reviewed in [28]), suggesting that occasional spillover of ABLV into other animals is likely. Thus, a major question regarding ABLV is its potential for cross-species transmission in terrestrial species other than humans and horses. We recently sought to address this question by defining the host cell infection tropism of ABLV. In agreement with the broad *in vitro* tropism reported for RABV [29], we found that numerous cell lines derived from several different mammalian species, including multiple species of small rodents, rabbit, human, monkey, and horse, were permissive to viral entry mediated by the G glycoproteins of both ABLV variants [30]. These results indicate that the ABLV host cell receptor is broadly conserved among mammals and suggests that species other than bats, humans, and horses could potentially be susceptible to ABLV infection. At present, the risk of human exposure to ABLV is currently believed to be limited to contact with infected bats; however, the recent horse infections demonstrate that other animals could potentially pose a threat. Furthermore, establishment of ABLV in terrestrial species would significantly increase this risk. Molecular evidence suggests that terrestrial RABV evolved from bat lyssaviruses [31], with dogs being one of the major terrestrial reservoirs of RABV. As recently as 2001, a bat variant of RABV emerged that successfully adapted to skunks [32]. Experimental infections of ABLV in terrestrial species are limited. Dogs and cats experimentally infected with a laboratory adapted strain of ABLVp exhibited mild behavioral changes and seroconverted within the three-month study, but none succumbed to ABLV and no viral antigen was detected at necropsy [33]. However, this study was only carried out for three months and it is possible that this was not sufficient time for the virus to reach the brain; one of the documented human ABLVp infections had an incubation period of more than two years [21]. The ability of ABLVs to cause clinical disease in dogs and cats has not been evaluated; however, given the recent spillover of ABLVs into horses and *in vitro* data demonstrating that cat embryo cells are significantly more susceptible to ABLVs G- than to ABLVp G-mediated infection [30], it is quite possible that clinical outcomes of dogs and cats inoculated with ABLVs would be much more severe. Further work is needed to fully define the potential risk of ABLV transmission and possible adaptation to terrestrial species.

## 4. Molecular Biology

### 4.1. Taxonomy

ABLV is a member of the family *Rhabdoviridae*, genus *Lyssavirus*; all lyssaviruses are capable of causing fatal acute encephalitis indistinguishable from clinical rabies in humans and other mammals. There are currently 12 classified species (genotypes) of lyssaviruses: *Rabies virus* (RABV), *Lagos bat virus* (LBV), *Mokola virus* (MOKV), *Duvenhage virus* (DUVV), *European bat lyssavirus* types 1 and 2 (EBLV-1 and -2), *Australian bat lyssavirus* (ABLV), *Aravan virus* (ARAV), *Khujand virus* (KHUV), *Irkut virus* (IRKV), *West Caucasian bat virus* (WCBV), and *Shimoni bat virus* (SHIBV). Three more recently described bat lyssaviruses have not yet been officially classified as distinct genotypes: Bokeloh bat lyssavirus (BBLV), Ikoma lyssavirus (IKOV), and Lleida bat lyssavirus (LLEBV) [34]. Lyssaviruses are also separated into three phylogroups, based on their genetic, immunologic, and pathogenic characteristics. Phylogroup I includes RABV, DUVV, EBLV-1, EBLV-2, ABLV, ARAV, IRKV, BBLV, and KHUV, phylogroup II includes LBV, MOKV, and SHIBV, and phylogroup III includes WCBV, IKOV, and LLEBV (Table 1) [34]. With the exception of Mokola virus and Ikoma lyssavirus, all lyssavirus species have known bat reservoirs, leading to the speculation that lyssaviruses originated in the order Chiroptera [31]. Human clinical rabies cases have been documented for RABV, MOKV, DUVV, EBLV-1, EBLV-2, ABLV, and IRKV (reviewed in [28]).

**Table 1:** Genotype and phylogroup classifications for the lyssaviruses. NC, not classified; these viruses are not yet classified as distinct lyssavirus species (genotypes) according to the International Committee on the Taxonomy of Viruses (ICTV) [35]. *, Human cases have been documented.

Lyssaviruses	Genotype	Phylogroup
* Rabies virus (RABV)	1	I
Lagos Bat virus (LBV)	2	II
* Mokola virus (MOKV)	3	II
* Duvenhage virus (DUVV)	4	I
* European bat lyssavirus 1 (EBLV1)	5	I
* European bat lyssavirus 2 (EBLV2)	6	I
* Australian bat lyssavirus (ABLV)	7	I
Aravan virus (ARAV)	8	I
Khujand virus (KHUV)	9	I
* Irkut virus (IRKV)	10	I
West Caucasian bat virus (WCBV)	11	III
Shimoni bat virus (SHIBV)	12	III
Bokeloh bat lyssavirus (BBLV)	NC	I
Ikoma lyssavirus (IKOV)	NC	III
Lleida bat lyssavirus (LLEBV)	NC	III

### 4.2. Lyssavirus Virion Structure

Lyssaviruses are enveloped, bullet-shaped viruses with a single-stranded, negative sense RNA genome of about 12 kb that encodes five viral proteins: nucleoprotein (N), phosphoprotein (P), matrix (M), glycoprotein (G), and RNA polymerase (L). The RNA genome is encapsidated by the N protein, forming the ribonucleoprotein (RNP) complex. Only the RNP is a functional template for transcription and replication. The L and P proteins associate with the RNP, forming the viral capsid. A host cell derived membrane surrounds the viral capsid and is associated with the M and G glycoproteins. The M protein serves as a bridge between the viral capsid and the virion membrane. The G glycoproteins associate into trimers on the virion surface and mediate viral attachment to and fusion with the host cell membrane [36]. Following host cell attachment, lyssaviruses are internalized by means of receptor-mediated endocytosis; the low pH of the endosome triggers G-mediated fusion of the viral and host cell membranes. 

## 5. Lyssavirus Entry

### 5.1. Attachment to Host Cells

The first step of the lyssavirus life cycle is attachment to the host cell which is mediated by the G glycoprotein; however, it is unclear which host cell surface molecule(s) interacts with the G glycoprotein to mediate viral entry into the cell. Lyssaviruses most likely enter motor neurons at the neuromuscular junction [37]; however, a study using a bat-derived RABV variant demonstrated that the virus was able to spread in the blood and enter the CNS at the neurovascular junction of the hypothalamus [38]. Numerous host cell molecules have been proposed as receptors for RABV, but none have been shown to be essential *in vitro*. These include protein receptors such as nicotinic acetylcholine receptor (nAchR) [39,40,41], neuronal cell adhesion molecule (NCAM) [29], and p75 neurotrophin receptor (p75NTR) [42], as well as neuraminic acid containing glycolipids (gangliosides) [43,44]. The nAchR is predominantly expressed in muscle cells and is localized to the postsynaptic muscle membrane, not to the presynaptic nerve membrane [45]; thus, it is unlikely that initial entry into motor neurons occurs through this receptor. While nAchR is not required for RABV infection of neurons [46], it may account for replication of RABV in muscle cells at the site of inoculation [47]. Although expression of p75NTR was sufficient to permit entry of a RABV fox field isolate into nonpermissive cells [42], it does not bind to most lyssavirus G glycoproteins, including ABLV G [48], and is not essential for RABV infection [49]. Similarly, although NCAM was shown to be an *in vitro* receptor for a fixed strain of RABV, NCAM knock-out mice were still susceptible to infection by RABV, although clinical onset was delayed by a few days [29].

The contribution of proposed RABV receptors to the entry of other lyssaviruses is largely unknown. We recently identified cell lines that are resistant to ABLV G-mediated viral entry [30]. One such cell line, CHOK1 cells, were previously reported to be highly permissive to the CVS-11 strain of RABV [29], suggesting that ABLV and RABV may utilize alternate receptors for host cell entry. We confirmed the high susceptibility of CHOK1 cells to RABV CVS-11 G-mediated viral entry and also found that PCI-13 cells, another ABLV resistant line, were also highly permissive to RABV G-mediated entry [30]. Both CHOK1 and PCI-13 cells were shown to express the proposed RABV protein receptors nAchR and NCAM, indicating that nAchR and NCAM are not sufficient to allow host cell entry of ABLV. Moreover, HeLa-USU cells, which do not express NCAM on the cell surface, are permissive to ABLV G-mediated infection further demonstrating that NCAM is not essential for ABLV infection [30]. The nature of the ABLV receptor(s) has not yet been determined; however, ABLV G-mediated entry was significantly reduced following lipid raft disruption with the cholesterol sequestering drug methyl-β-cyclodextrin [30]. Taken together, these results suggest that ABLV utilizes a unique receptor or co-receptor for host cell infection that is localized or enriched in lipid rafts. 

Thus, although ABLV and RABV are closely related and are capable of causing similar fatal neurological disease in humans, they display unique *in vitro* tropisms and can utilize alternate host receptors for viral entry. Interestingly, we found that the same is true for *Saccolaimus* and *Pteropus* variants of ABLV which showed a 6 to 45-fold difference in infectivity in three different cell lines derived from three different species [30]. These recent findings highlight the need for further research on lyssavirus entry. The ability of most fixed strains of RABV to replicate in most continuous cell lines [29,41] has hampered RABV receptor discovery since methods that have been successfully used to identify receptors for other viruses could not be used for RABV. The recent identification of ABLV resistant cells [30] will provide a platform for identifying a lyssavirus receptor that has been lacking until now. ABLV resistant CHOK1 cells in particular, which are easy to transfect with expression plasmids, will be an invaluable tool for future studies aimed at ABLV receptor identification.

### 5.2. Internalization

Following host cell attachment, lyssaviruses are internalized into the host cell by receptor-mediated endocytosis. Pinocytic mechanisms of endocytosis, which permit the uptake of fluid, small particles, and solutes into the host cell, are typically exploited by viruses to gain entry into cells. Clathrin-mediated endocytosis (CME), caveolin/raft-dependent endocytosis (CavME), and macropinocytosis are the best studied endocytic mechanisms, but there are several clathrin- and caveolin/raft-independent mechanisms that are less understood. The CME pathway is the most commonly utilized pathway in virus entry, but there are examples of viruses using at least six additional endocytic pathways for host cell entry (reviewed in [50]). Numerous methodologies including imaging, chemical inhibition, and dominant negative molecular approaches have demonstrated that both RABV and ABLV enter cells via a clathrin- and dynamin-dependent pathway [51,52,53]. Rhabdoviruses from other genera also utilize CME for host cell entry, including vesicular stomatitis virus (VSV) (genus *Vesiculovirus*) [54] and infectious hematopoietic necrosis virus (IHNV) (genus *Novirhabdovirus*) [55]; to date no rhabdovirus has been reported to utilize a clathrin-independent pathway. This appears to also be the case for ABLV as chemical inhibition of CavME and macropinocytosis did not reduce ABLV G-mediated viral entry into HEK293T cells [53]. 

CME is initiated through the virus-host cell receptor interaction which induces *de novo* clathrin-coated pit (CCP) formation at the site of viral binding [56,57,58,59]. For RABV and VSV, the CCP assembly phase lasts about twice as long as normal CCP formation (110 s *versus* 50 s, respectively) [52,57,59]. Though it is not clear what causes clathrin-coat assembly, it has been postulated that receptor clustering induced by surface bound virus may cause the formation of a microdomain with differing properties than that of the surrounding membrane [50]. Both, RABV and VSV, have been shown to be internalized by pits partially coated with clathrin that require actin polymerization for internalization; this dependence on actin is dictated by the size of the particle, as truncated defective-interfering particles of VSV do not require actin [52,59,60]. We recently found that ABLV entry is also actin-dependent [53], as is the entry of the IHNV [55]. Given the shared particle morphology of all rhabdoviruses, it is likely that the entry of other rhabdovirus species is also actin-dependent. The mechanism by which cell surface bound viral particles induce actin recruitment to the CCPs is not completely understood. However, recent studies have demonstrated that plasma membrane tension induces the actin dependence of clathrin coat assembly. It has been postulated that upon cell surface binding, the viral particles themselves induce membrane tension, thus leading to the recruitment of actin to the forming clathrin-coated vesicle [52,59,60,61]. After invagination, the clathrin-coated vesicle is pinched from the membrane by dynamin, a self-polymerizing GTPase [62,63,64]. Clathrin lattices are then disassembled from the detached vesicle, enabling the uncoated vesicle to traffic to and fuse with its target endosome [65].

### 5.3. Uncoating

Once internalized by endocytosis, viruses follow the same intracellular vesicular trafficking pathways as physiological ligands and membrane components, such as hormones, growth factors, and plasma membrane factors. These endosomal systems are responsible for molecular sorting, recycling, degradation, and transcytosis of incoming cargo. The primary organelles in the endosomal network are early endosomes (EEs) (pH 6.5–6.0), maturing endosomes (MEs) (pH 6.0), late endosomes (LEs) (pH 6.0–5.0), recycling endosomes (REs), and lysosomes [50]. The low-pH of the endosome triggers a conformational change in the viral glycoprotein spike that facilitates viral and endosomal membrane fusion and the subsequent release of the viral genome into the cytosol. The pH required to trigger fusion typically correlates with the pH of the endosome.

Viruses delivered from the surface by CME are typically delivered to EEs in less than two minutes and to the LEs within 10–12 min, with viral and host membrane fusion taking place within 1–5 min (VSV) or 10–20 min (influenza and dengue viruses), respectively [50,66,67,68,69]. Selective vesicular trafficking and sorting is determined by functionally distinct domains localized on the cytosolic face of the endosomal membrane that are defined by different Rab GTPases and their effectors. In their active states, Rab proteins are GTP-bound and are recognized by multiple effector proteins; in inactive states they are GDP-bound. A guanine exchange factor (GEF) catalyzes the GDP to GTP exchange. The active Rab is converted back to the GDP-bound inactive state by the release of an inorganic phosphate by GTPase-activating protein (GAP) [70]. Active Rab GTPases coordinate cargo trafficking within the endosomal network. For example, clathrin-coated vesicles are transported to EEs through the interactions of Rab5 (a marker of EEs) with AP2 to facilitate the uncoating of clathrin-coated vesicles and subsequent fusion with EEs [71]. Once delivered to the EEs, the Rab defined domains either target cargo back to the plasma membrane (Rab4), to LEs (Rab7), to REs (Rab22/Rab11), or to the *trans*-Golgi network (Rab9) [50].

Lyssaviruses require vacuolar acidification for G glycoprotein-mediated viral fusion with host cell membranes [72], but few studies have examined the endosomal trafficking of these viruses. RABV G was shown to co-localize with Rab5a (EEs) but not Rab9 (another marker of LEs); this same study demonstrated that internalization of an anti-RABV G antibody/RABV G complex was dependent on the presence of functional Rab5a suggesting that RABV G fuses with EEs [73]. Separate studies showed that RABV co-localized with endocytic tracers for early endosomes within nerve cells [74,75]. Furthermore, the optimal pH for RABV G-mediated fusion is pH 5.8–6.0, which correlates with the pH of EEs [50,76]. Dominant negative forms of Rab5, Rab7, and Rab11 were recently used to investigate the endosomal trafficking of ABLV G. ABLV G-mediated viral entry was found to be dependent on Rab5 but not on Rab7 or Rab11, indicating that similar to RABV G, ABLV G likely fuses with early endosomal membranes to gain entry into the host cell cytosol [53]. Further supporting this argument, similar to that of RABV G, ABLV G-mediated fusion is optimal between pH 5.7–5.9 [77].

### 5.4. Model for Lyssavirus Entry

**Figure 2:**
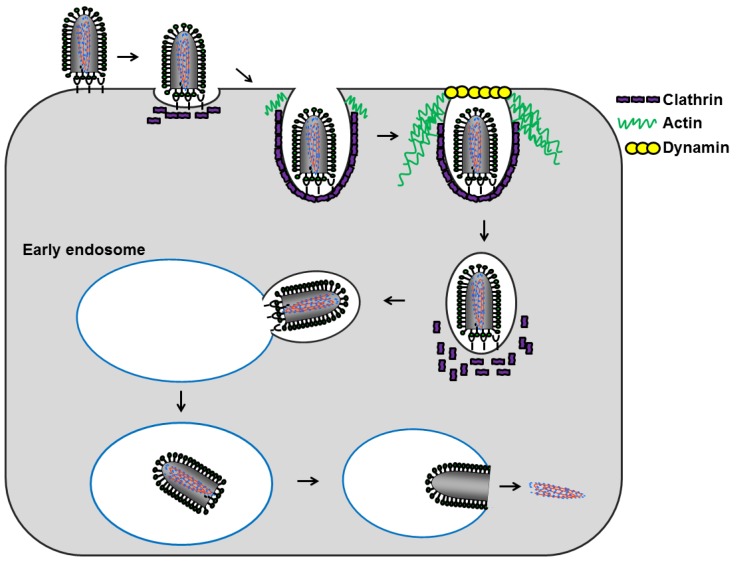
Lyssavirus host cell entry. Following attachment to host cell receptors, the virus particles are endocytosed via a clathrin- and dynamin- dependent pathway that requires actin polymerization for complete vesicle envelopment. The internalized uncoated vesicle then traffics to and fuses with the early endosome. The mildly acidic environment of the endosome triggers conformational changes within G that permits fusion of the viral and endosomal membranes and subsequent release of the nucleocapsid into the cytoplasm.

Lyssavirus infection occurs at the nervous system periphery following a bite or scratch from an infected animal. Following initial entry into motor neurons at the neuromuscular junction [37], the virus is transported via retrograde axonal transport [78] to the neuronal cell body which provides an environment conducive for protein synthesis [79]. Two different models have been proposed for the transport of RABV: either viral and host membrane fusion occurs shortly after entry and the nucleocapsid is transported alone to the cell body or the whole virion is transported inside a vesicle and fusion occurs within the cell body (recently reviewed in [80,81]). Regardless of when the fusion event takes place, lyssaviruses appear to primarily follow a clathrin-and dynamin- dependent entry pathway that requires actin for productive infection. The mildly acidic environment characteristic of EEs is sufficient to trigger G-mediated viral and endosomal membrane fusion and subsequent release of the viral genome into the cytosol (Figure 2).

## 6. Conclusions

Australia was one of the few countries considered free of endemic lyssaviruses until the discovery of ABLV in 1996. The fatal human infections and the recent horse infections have made ABLV a considerable health concern to wildlife, veterinary, and healthcare officials. RABV post-exposure prophylaxis (PEP) is currently used to treat humans that are exposed to bats suspected of being infected with ABLV and consists of the administration of human rabies immune globulin (HRIG) and a four dose rabies vaccine series [82]. While rabies vaccination was shown to provide complete cross-protection of mice challenged intracerebrally with the *Pteropus* variant (ABLVp) [6], a separate study found that of RABV vaccinated mice challenged with the *Saccolaimus* variant (ABLVs), only 50% and 79% survived intracranial and peripheral challenge, respectively [83]. Virus titration studies in mice have revealed that ABLVs may have a shorter incubation time in mice compared to ABLVp [84], which could make successful vaccination against ABLVs more challenging. Further research is needed to evaluate the effectiveness of rabies vaccines to protect against ABLVs infection.

There is no effective treatment for disease caused by ABLV or other lyssaviruses once symptoms begin. Like all viruses, lyssaviruses rely on the host cell machinery for replication; thus, understanding virus-host cell interactions, such as virus attachment to host cells, receptor-mediated endocytosis, and uncoating, provides a means for identifying potential targets for antiviral therapeutics. Recent studies have highlighted that although ABLV and RABV exhibit unique tropisms and can utilize alternate receptors for host cell virus entry [30], they appear to utilize similar entry pathways [53]; this suggests that the identification of drug targets for the development of broad spectrum lyssavirus antiviral therapeutics may be possible. However, given that the host factors that have been thus far identified as being required for ABLV and RABV entry, such as clathrin, dynamin, actin, and Rab5, are all critical for host cellular processes, they may not make viable antiviral drug targets. Ongoing studies by our group are focused on identifying additional host factors required for ABLV/lyssavirus infection.

The recent human and horse infections highlight the need for continued and improved public health awareness of ABLV. Neither the latest ABLV victim nor his family was aware of the potential health risks associated with exposure to bats [23]. If they had been aware, then perhaps administration of PEP would have prevented the fatal outcome. The recent spillover of ABLVs into horses and *in vitro* ABLV tropism analyses indicate that animals other than bats could pose potential health threats for ABLV exposure of people. Further work is needed to fully define the cross-species transmission potential of ABLV.

## Supplementary Files

Supplementary File 1 Supplementary Materials (ZIP, 24713 KB)

## References

[1] Murray K., Selleck P., Hooper P., Hyatt A., Gould A., Gleeson L., Westbury H., Hiley L., Selvey L., Rodwell B. (1995). A morbillivirus that caused fatal disease in horses and humans. Science.

[2] Young P.L., Halpin K., Selleck P.W., Field H., Gravel J.L., Kelly M.A., Mackenzie J.S. (1996). Serologic evidence for the presence in Pteropus bats of a paramyxovirus related to equine morbillivirus. Emerg. Infect. Dis..

[3] Halpin K., Young P.L., Field H.E., Mackenzie J.S. (2000). Isolation of Hendra virus from pteropid bats: A natural reservoir of Hendra virus. J. Gen. Virol..

[4] Fraser G.C., Hooper P.T., Lunt R.A., Gould A.R., Gleeson L.J., Hyatt A.D., Russell G.M., Kattenbelt J.A. (1996). Encephalitis caused by a Lyssavirus in fruit bats in Australia. Emerg. Infect. Dis..

[5] Gould A.R., Hyatt A.D., Lunt R., Kattenbelt J.A., Hengstberger S., Blacksell S.D. (1998). Characterisation of a novel lyssavirus isolated from Pteropid bats in Australia. Virus Res..

[6] Hooper P.T., Lunt R.A., Gould A.R., Samaratunga H., Hyatt A.D., Gleeson L.J., Rodwell B.J., Rupprecht C.E., Smith J.S., Murray P.K. (1997). A new lyssavirus: The first endemic rabies-related virus recognized in Australia. Bull. Inst. Pasteur..

[7] Gould A.R., Kattenbelt J.A., Gumley S.G., Lunt R.A. (2002). Characterisation of an Australian bat lyssavirus variant isolated from an insectivorous bat. Virus Res..

[8] Wilson D.E., Reeder D.M. (2005). Mammal Species of the World. A Taxonomic and Geographic Reference.

[9] Hall L., Richards G. (2000). Flying Foxes: Fruit and Blossom Bats of Australia.

[10] McCall B.J., Epstein J.H., Neill A.S., Heel K., Field H., Barrett J., Smith G.A., Selvey L.A., Rodwell B., Lunt R. (2000). Potential exposure to Australian bat lyssavirus, Queensland, 1996–1999. Emerg. Infect. Dis..

[11] Arguin P.M., Murray-Lillibridge K., Miranda M.E., Smith J.S., Calaor A.B., Rupprecht C.E. (2002). Serologic evidence of Lyssavirus infections among bats, the Philippines. Emerg. Infect. Dis..

[12] Van Dyke S., Strahan R. (2008). The Mammals of Australia.

[13] Animal Health Australia (2013). Response Policy Brief: Hendra Virus Infection (Version 3.5). Australian Veterinary Emergency Plan (AUSVETPLAN).

[14] Richards G., Hall L. (2012). A Natural History of Australian Bats: Working the Night Shift.

[15] Field H.E. (2005). Australian Bat Lyssavirus. Ph.D. Thesis.

[16] Barrett J. (2004). Australian Bat Lyssavirus. Ph.D. Thesis.

[17] Field H., McCall B., Barrett J. (1999). Australian bat lyssavirus infection in a captive juvenile black flying fox. Emerg. Infect. Dis..

[18] Warrilow D., Harrower B., Smith I.L., Field H., Taylor R., Walker C., Smith G.A. (2003). Public health surveillance for Australian bat lyssavirus in Queensland, Australia, 2000–2001. Emerg. Infect. Dis..

[19] Allworth A., Murray K., Morgan J. (1996). A human case of encephalitis due to a lyssavirus recently identified in fruit bats. Commun. Dis. Intellig..

[20] Samaratunga H., Searle J.W., Hudson N. (1998). Non-rabies Lyssavirus human encephalitis from fruit bats: Australian bat Lyssavirus (pteropid Lyssavirus) infection. Neuropathol. Appl. Neurobiol..

[21] Hanna J.N., Carney I.K., Smith G.A., Tannenberg A.E., Deverill J.E., Botha J.A., Serafin I.L., Harrower B.J., Fitzpatrick P.F., Searle J.W. (2000). Australian bat lyssavirus infection: A second human case, with a long incubation period. Med. J. Aust..

[22] Australian bat lyssavirus—Australia (02). http://www.promedmail.org/direct.php?id=20130323.1600266.

[23] Hayes L. Australian Rabies. http://sixtyminutes.ninemsn.com.au/article.aspx?id=8667746.

[24] Arthur R., Human F., Williamson G., Dickason C., Conway M.L., Bell C., Finkelstein J. (2013). State and territory reports. Animal Health Surveillance Quarterly Report.

[25] Roth I. (2013). CVO Bulletin to NSW Veterinarians regarding Australian Bat Lyssavirus (ABLV).

[26] Annand E.A., Shinwari W. (2014).

[27] McCall B.J., Field H.E., Smith G.A., Storie G.J., Harrower B.J. (2005). Defining the risk of human exposure to Australian bat lyssavirus through potential non-bat animal infection. Commun. Dis. Intell..

[28] Banyard A.C., Hayman D., Johnson N., McElhinney L., Fooks A.R. (2011). Bats and lyssaviruses. Adv. Virus Res..

[29] Thoulouze M.I., Lafage M., Schachner M., Hartmann U., Cremer H., Lafon M. (1998). The neural cell adhesion molecule is a receptor for rabies virus. J. Virol..

[30] Weir D.L., Smith I.L., Bossart K.N., Wang L.F., Broder C.C. (2013). Host cell tropism mediated by Australian bat lyssavirus envelope glycoproteins. Virology.

[31] Badrane H., Tordo N. (2001). Host switching in Lyssavirus history from the Chiroptera to the Carnivora orders. J. Virol..

[32] Leslie M.J., Messenger S., Rohde R.E., Smith J., Cheshier R., Hanlon C., Rupprecht C.E. (2006). Bat-associated rabies virus in Skunks. Emerg. Infect. Dis..

[33] McColl K.A., Chamberlain T., Lunt R.A., Newberry K.M., Westbury H.A. (2007). Susceptibility of domestic dogs and cats to Australian bat lyssavirus (ABLV). Vet. Microbiol..

[34] Ceballos N.A., Moron S.V., Berciano J.M., Nicolas O., Lopez C.A., Juste J., Nevado C.R., Setien A.A., Echevarria J.E. (2013). Novel lyssavirus in bat, Spain. Emerg. Infect. Dis..

[35] Virus Taxonomy: 2012 release. http://www.ictvonline.org/virusTaxonomy.asp.

[36] Gaudin Y., Ruigrok R.W., Tuffereau C., Knossow M., Flamand A. (1992). Rabies virus glycoprotein is a trimer. Virology.

[37] Watson H.D., Tignor G.H., Smith A.L. (1981). Entry of rabies virus into the peripheral nerves of mice. J. Gen. Virol..

[38] Preuss M.A., Faber M.L., Tan G.S., Bette M., Dietzschold B., Weihe E., Schnell M.J. (2009). Intravenous inoculation of a bat-associated rabies virus causes lethal encephalopathy in mice through invasion of the brain via neurosecretory hypothalamic fibers. PLoS Pathog..

[39] Lentz T.L., Burrage T.G., Smith A.L., Crick J., Tignor G.H. (1982). Is the acetylcholine receptor a rabies virus receptor?. Science.

[40] Lentz T.L., Wilson P.T., Hawrot E., Speicher D.W. (1984). Amino acid sequence similarity between rabies virus glycoprotein and snake venom curaremimetic neurotoxins. Science.

[41] Hanham C.A., Zhao F., Tignor G.H. (1993). Evidence from the anti-idiotypic network that the acetylcholine receptor is a rabies virus receptor. J. Virol..

[42] Tuffereau C., Benejean J., Blondel D., Kieffer B., Flamand A. (1998). Low-affinity nerve-growth factor receptor (P75NTR) can serve as a receptor for rabies virus. EMBO J..

[43] Superti F., Hauttecoeur B., Morelec M.J., Goldoni P., Bizzini B., Tsiang H. (1986). Involvement of gangliosides in rabies virus infection. J. Gen. Virol..

[44] Conti C., Superti F., Tsiang H. (1986). Membrane carbohydrate requirement for rabies virus binding to chicken embryo related cells. Intervirology.

[45] Lafon M. (2005). Rabies virus receptors. J. Neurovirol..

[46] McGehee D.S., Role L.W. (1995). Physiological diversity of nicotinic acetylcholine receptors expressed by vertebrate neurons. Annu. Rev. Physiol..

[47] Burrage T.G., Tignor G.H., Smith A.L. (1985). Rabies virus binding at neuromuscular junctions. Virus Res..

[48] Tuffereau C., Desmezieres E., Benejean J., Jallet C., Flamand A., Tordo N., Perrin P. (2001). Interaction of lyssaviruses with the low-affinity nerve-growth factor receptor p75NTR. J. Gen. Virol..

[49] Tuffereau C., Schmidt K., Langevin C., Lafay F., Dechant G., Koltzenburg M. (2007). The rabies virus glycoprotein receptor p75NTR is not essential for rabies virus infection. J. Virol..

[50] Mercer J., Schelhaas M., Helenius A. (2010). Virus entry by endocytosis. Annu. Rev. Biochem..

[51] Superti F., Derer M., Tsiang H. (1984). Mechanism of rabies virus entry into CER cells. J. Gen. Virol..

[52] Piccinotti S., Kirchhausen T., Whelan S.P. (2013). Uptake of rabies virus into epithelial cells by clathrin-mediated endocytosis depends upon actin. J. Virol..

[53] Weir D.L., Laing E.D., Smith I.L., Wang L.F., Broder C.C. (2014). Host cell virus entry mediated by Australian bat lyssavirus G envelope glycoprotein occurs through a clathrin-mediated endocytic pathway that requires actin and Rab5. Virol. J..

[54] Sun X., Yau V.K., Briggs B.J., Whittaker G.R. (2005). Role of clathrin-mediated endocytosis during vesicular stomatitis virus entry into host cells. Virology.

[55] Liu H., Liu Y., Liu S., Pang D.W., Xiao G. (2011). Clathrin-mediated endocytosis in living host cells visualized through quantum dot labeling of infectious hematopoietic necrosis virus. J. Virol..

[56] Ehrlich M., Boll W., van Oijen A., Hariharan R., Chandran K., Nibert M.L., Kirchhausen T. (2004). Endocytosis by random initiation and stabilization of clathrin-coated pits. Cell.

[57] Johannsdottir H.K., Mancini R., Kartenbeck J., Amato L., Helenius A. (2009). Host cell factors and functions involved in vesicular stomatitis virus entry. J. Virol..

[58] Rust M.J., Lakadamyali M., Zhang F., Zhuang X. (2004). Assembly of endocytic machinery around individual influenza viruses during viral entry. Nat. Struct. Mol. Biol..

[59] Cureton D.K., Massol R.H., Saffarian S., Kirchhausen T.L., Whelan S.P. (2009). Vesicular stomatitis virus enters cells through vesicles incompletely coated with clathrin that depend upon actin for internalization. PLoS Pathog..

[60] Cureton D.K., Massol R.H., Whelan S.P., Kirchhausen T. (2010). The length of vesicular stomatitis virus particles dictates a need for actin assembly during clathrin-dependent endocytosis. PLoS Pathog..

[61] Boulant S., Kural C., Zeeh J.C., Ubelmann F., Kirchhausen T. (2011). Actin dynamics counteract membrane tension during clathrin-mediated endocytosis. Nat. Cell Biol..

[62] Hinshaw J.E., Schmid S.L. (1995). Dynamin self-assembles into rings suggesting a mechanism for coated vesicle budding. Nature.

[63] Sweitzer S.M., Hinshaw J.E. (1998). Dynamin undergoes a GTP-dependent conformational change causing vesiculation. Cell.

[64] Bashkirov P.V., Akimov S.A., Evseev A.I., Schmid S.L., Zimmerberg J., Frolov V.A. (2008). GTPase cycle of dynamin is coupled to membrane squeeze and release, leading to spontaneous fission. Cell.

[65] Ungewickell E., Ungewickell H., Holstein S.E., Lindner R., Prasad K., Barouch W., Martin B., Greene L.E., Eisenberg E. (1995). Role of auxilin in uncoating clathrin-coated vesicles. Nature.

[66] Blumenthal R., Bali-Puri A., Walter A., Covell D., Eidelman O. (1987). pH-dependent fusion of vesicular stomatitis virus with Vero cells. Measurement by dequenching of octadecyl rhodamine fluorescence. J. Biol. Chem..

[67] Van der Schaar H.M., Rust M.J., Waarts B.L., van der Ende-Metselaar H., Kuhn R.J., Wilschut J., Zhuang X., Smit J.M. (2007). Characterization of the early events in dengue virus cell entry by biochemical assays and single-virus tracking. J. Virol..

[68] Lakadamyali M., Rust M.J., Zhuang X. (2004). Endocytosis of influenza viruses. Microbes Infect..

[69] Lakadamyali M., Rust M.J., Zhuang X. (2006). Ligands for clathrin-mediated endocytosis are differentially sorted into distinct populations of early endosomes. Cell.

[70] Stenmark H. (2009). Rab GTPases as coordinators of vesicle traffic. Nat. Rev. Mol. Cell Biol..

[71] Semerdjieva S., Shortt B., Maxwell E., Singh S., Fonarev P., Hansen J., Schiavo G., Grant B.D., Smythe E. (2008). Coordinated regulation of AP2 uncoating from clathrin-coated vesicles by rab5 and hRME-6. J. Cell Biol..

[72] Mifune K., Ohuchi M., Mannen K. (1982). Hemolysis and cell fusion by rhabdoviruses. FEBS Lett..

[73] St Pierre C.A., Leonard D., Corvera S., Kurt-Jones E.A., Finberg R.W. (2011). Antibodies to cell surface proteins redirect intracellular trafficking pathways. Exp. Mol. Pathol..

[74] Lewis P., Fu Y., Lentz T.L. (1998). Rabies virus entry into endosomes in IMR-32 human neuroblastoma cells. Exp. Neurol..

[75] Lewis P., Lentz T.L. (1998). Rabies virus entry into cultured rat hippocampal neurons. J. Neurocytol..

[76] Gaudin Y., Ruigrok R.W., Knossow M., Flamand A. (1993). Low-pH conformational changes of rabies virus glycoprotein and their role in membrane fusion. J. Virol..

[77] Weir D.L. (2014).

[78] Ugolini G. (1995). Specificity of rabies virus as a transneuronal tracer of motor networks: Transfer from hypoglossal motoneurons to connected second-order and higher order central nervous system cell groups. J. Comp. Neurol..

[79] Malgaroli A., Vallar L., Zimarino V. (2006). Protein homeostasis in neurons and its pathological alterations. Curr. Opin. Neurobiol..

[80] Schnell M.J., McGettigan J.P., Wirblich C., Papaneri A. (2010). The cell biology of rabies virus: Using stealth to reach the brain. Nat. Rev. Microbiol..

[81] Albertini A.A., Baquero E., Ferlin A., Gaudin Y. (2012). Molecular and cellular aspects of rhabdovirus entry. Viruses.

[82] (2012). CDNA National Guidelines for Public Health Units: Rabies Virus and Other Lyssavirus (Including Australian Bat Lyssavirus) Exposures and Infections.

[83] Brookes S.M., Parsons G., Johnson N., McElhinney L.M., Fooks A.R. (2005). Rabies human diploid cell vaccine elicits cross-neutralising and cross-protecting immune responses against European and Australian bat lyssaviruses. Vaccine.

[84] Moore P.R. (2011). Characterisation of Australian Bat Lyssavirus and an Evaluation of the Rabies Vaccines. Ph.D. Thesis.

